# Biological Relevance of Extra Virgin Olive Oil Polyphenols Metabolites

**DOI:** 10.3390/antiox7120170

**Published:** 2018-11-22

**Authors:** Gabriele Serreli, Monica Deiana

**Affiliations:** Department of Biomedical Sciences, University of Cagliari, Cittadella Universitaria, SS 554, km 4.5, 09042 Monserrato, Italy; gabriele.serreli@unica.it

**Keywords:** hydroxytyrosol, tyrosol, extra virgin olive oil, polyphenols, metabolites, homovanillyl alcohol, homovanillic acid

## Abstract

Extra virgin olive oil (EVOO) polyphenols beneficial effects have widely been debated throughout the last three decades, with greater attention to hydroxytyrosol and tyrosol, which are by far the most studied. The main concern about the evaluation of EVOO phenols activities in vitro and in vivo is that the absorption and metabolism of these compounds once ingested lead to the production of different metabolites in the human body. EVOO phenols in the ingested forms are less concentrated in human tissues than their glucuronide, sulfate and methyl metabolites; on the other hand, metabolites may undergo deconjugation before entering the cells and thus act as free forms or may be reformed inside the cells so acting as conjugated forms. In most in vitro studies the presence of methyl/sulfate/glucuronide functional groups does not seem to inhibit biological activity. Parent compounds and metabolites have been shown to reach tissue concentrations useful to exert beneficial effects others than antioxidant and scavenging properties, by modulating intracellular signaling and improving cellular response to oxidative stress and pro-inflammatory stimuli. This review aims to give an overview on the reported evidence of the positive effects exerted by the main EVOO polyphenols metabolites in comparison with the parent compounds.

## 1. Introduction

The consumption of extra virgin olive oil (EVOO) is feature of the Mediterranean Diet, which has been largely associated to several health benefits [1,2,3,4]. It is well established that these beneficial effects are partly due to EVOO content of hydrophilic components such as polyphenols, which are known for their remarkable antioxidant activity [5,6]. Main EVOO polyphenols hydroxytyrosol (2-(3,4-dihydroxyphenyl)ethanol, HT) and tyrosol (2-(4-hydroxyphenyl)ethanol, Tyr) and other less concentrated phenolic compounds are important at biological level not only for their antioxidant activity [7], but also for their implication in the modulation of several intracellular signals [2,6,8,9,10] which is reflected in other beneficial effects than scavenging free radicals. Indeed, EVOO polyphenols are well-known for their anti-inflammatory effects, exerted by down-regulating inflammatory mediators by transcriptional or post-transcriptional mechanisms and by modulating the activation of kinases involved in the onset of inflammatory process at different levels [2,11]. In addition, anti-cancer properties of EVOO polyphenols have been related to their interactions with proteins controlling cell cycle progression and gene expression [2,8,11,12,13]. 

Their effectiveness in vivo, however, should be carefully evaluated taking into account the absorption and metabolism kinetics of these compounds once ingested. In fact, it is known that Tyr and HT are exposed to an extensive metabolism in the human body, and their bioavailability is poor with respect to their metabolites [6]. Therefore, the major critical issue in relation to the studies on these molecules, especially those in vitro, is to consider the biological activity of the parent compounds, instead of their metabolites, which often are more concentrated in a specific tissue and could then exert more beneficial effects. In the last decade more and more research groups centered their studies on the evaluation of how and to which extent major metabolites of EVOO polyphenols were able to modulate intracellular signals and exert antioxidant and scavenging capacities. Trying to give an overview of these activities, the present review will focus on the biological significance of major EVOO polyphenols metabolites, considering their effect in different in vitro and in vivo studies in comparison with their parent compounds.

## 2. Olive Oil

Olive oil is one of the most important elements in the Mediterranean diet, not only for its appreciable taste and usefulness in flavoring a large variety of foods, but also for its several beneficial properties due to its chemical composition [11]. It is the main product obtained by olives, fruits which come from the Olea europaea evergreen trees [14]. This plant is being cultivated worldwide, but the Mediterranean area is the most favorable place where to grow it, because of its dry summers and mild winters [15]. In fact, the oil chemical composition and sensory properties depend on the climate, but also on the growing procedures, the cultivar of the olive, and the production techniques [16,17]. The oil extraction from olives is usually conducted through pressure, centrifugation, and percolation [15]. EVOO chemical composition is characterized by two major components: the saponifiables and the unsaponifiables compounds. The first group comprises triacylglycerols (TAG), partial glycerides, esters of fatty acids or free fatty acids and phosphatides, and represent nearly 98% of the oil chemical composition, while the second is formed by minor components such as hydrocarbons (squalene), phytosterols (β-sitosterol, stigmasterol, and campersterol), tocopherols, carotenoids, pigments (chlorophylls), aliphalic and triterpenic alcohols, triterpenic acids (oleanolic acid), volatile compounds and polyphenols. As regards the first group of compounds, the main acids present in olive oil are oleic (C18:1), palmitic (C16:0), palmitoleic (C16:1), stearic (C18:0), linoleic (C18:2), and linolenic (C18:3). Myristic (C14:0), heptadecanoic and eicosanoic acids are instead found in trace amounts. Among tocopherols, the level of the eight known “E-vitamers” in virgin olive oils has been shown to be almost composed by the α-homologue which comprises the 90% of the total tocopherol content and is found in the free form [14]. Chlorophylls are expressed mainly as pheophytin α and, to a lesser extent, as pheophytin β, while the main carotenoids found are lutein and β-carotene, followed by minor compounds such as violaxanthin, neoxanthin, luteoxanthin, antheraxanthin, mutatoxanthin, and β-cryptoxanthin. All of these compounds contribute, though differently, to the oil flavor and to its health benefits [14]. Countless studies have demonstrated the importance of the protective role of EVOO against the development of the most common degenerative diseases in the context of a balanced Mediterranean-type diet, and this beneficial effect has been ascribed both to its high monounsaturated fatty acids (MUFA) content and to the minor components, mainly to the phenolic fraction [18].

### 2.1. Extra-Virgin Olive Oil Polyphenols

The hydrophilic phenolic fraction, generally indicated as polyphenols, is only a small proportion of the numerous compounds which characterize EVOO composition. Nevertheless, EVOO polyphenols play a key role in the beneficial effects on human health attributed to EVOO. Indeed, they have been shown to possess antimicrobial, anticancer, antioxidant and anti-inflammatory properties, in vivo and in vitro [11,19]. The phenolic compounds are known to contribute to the sensory properties of EVOO [20]. They confer a bitter, pungent taste and a strong, fruity flavor to the oil, indicating a high sensory quality [21]. EVOOs with high phenol levels exhibit a high stability because of their antioxidant capacity, which prevents its autoxidation and contributes to determine a long shelf-life [22].

More than 30 phenolic compounds have been identified in EVOO, but not all of them are present in every oil [16]. The large variety of polyphenols found in EVOO is different in chemical structures and concentrations (0.02–600 mg/kg), depending on several factors including: variety, region in which the olive is grown, agricultural techniques, maturity of the olive fruit at harvest, and processing [16]. Indeed, different varieties, cultivated in the same environment and processed at a fixed ripening stage, produce EVOO with different total polyphenols content [23].

The main classes of EVOO polyphenols are the following:

- secoiridoids, where the most abundant are the dialdehydic form of decarboxymethyl elenolic acid linked to HT (3,4-DHPEA) or Tyr (p-HPEA), (3,4-DHPEA-EDA or p-HPEA-EDA), oleacein, oleuropein, an isomer of the oleuropein aglycon (HT linked to elenolic acid) (3,4-DHPEA-EA), and ligstroside aglycon (Tyr linked to elenolic acid) (p-HPEA-EA). p-HPEA-derivates and dialdehydic forms of oleuropein and ligstroside aglycon have also been detected as minor hydrophilic phenols of EVOO [19,24];
- phenylethanoids, which possess a hydroxyl group attached to an aromatic hydrocarbon group, such as oleocanthal, HT (3,4-dihydroxyphenyl-ethanol or 3,4-DHPEA) and Tyr (p-hydroxyphenyl-ethanol or p-HPEA) [6]. Their concentration is usually low in fresh oils but increases during oil storage due to the hydrolysis of secoiridoids [25];
- phenolic acids, which can be divided into two subgroups: hydroxybenzoic acid derivatives and hydroxycinnamic acid derivatives, such as gallic acid, protocatechuic acid, p-hydroxybenzoic acid, vanillic acid, caffeic acid, syringic acid, p- and o-coumaric acid, ferulic acid, and cinnamic acid [24];
- flavonoids, which contain two benzene rings joined by a linear three carbon chain. Flavonoids are largely planar molecules and their structural variation comes in part from the pattern of modification by hydroxylation, methoxylation, prenylation, or glycosylation. Flavonoid aglycones are subdivided into flavones, flavonols, flavanones, and flavanols depending upon the presence of a carbonyl carbon at C-4, an OH group at C-3, a saturated single bond between C-2 and C-3, and a combination of carbonyl at C-4 with an OH group at C-3, respectively. Luteolin and apigenin are the most concentrated [24];
- hydroxy-isocromans, a class which consists in only two compounds: 1-phenyl-6,7-dihydroxy-isochroman and 1-(39-methoxy-49-hydroxy) phenyl-6,7-dihydroxy- isochroman [26];
- lignans, whose structure is not well understood, but it is based on the condensation of aromatic aldehydes. (+)-1-Acetoxypinoresinol and (+)-1-pinoresinol were characterized as the most concentrated lignans in EVOO [27].

The phenolic acids were the first group of phenolic compounds found in EVOO; these compounds, together with phenyl-alcohols, hydroxy-isochromans and flavonoids [26], are present in small amounts in EVOO, while secoiridoids and lignans are the most prevalent ones [24]. 

### 2.2. Absorption and Distribution of Polyphenols

The degree to which phenolic compounds are bioavailable (absorbed, metabolized, distributed and eliminated) is fundamental in understanding and evaluating the health benefits associated with EVOO consumption; to achieve an effect in specific tissues or organs, in fact, they must be bioavailable [28]. The majority of researches regarding the bioavailability of these compounds have focused on the absorption and excretion of two major phenolics: HT and Tyr, and significant absorption (~40–95%) of these compounds has been demonstrated in humans [29]. The absorption and metabolism of other minor compounds have also been studied, such as hydroxycinnamic and hydroxybenzoic acid derivatives [30,31], and flavonoids [32,33]. The dietary intake of EVOO polyphenols in the Mediterranen diet, eating habits of the countries surrounding the Mediterranean Sea, has been estimated to be around 9 mg, within 25–50 mL of olive oil per day, where at least 1 mg of them is represented by free HT and Tyr, and 8 mg are related to their elenolic esters and to the oleuropein- and ligstroside-aglycons [5]. Concerning HT and Tyr, several clinical and animal studies have provided evidence that they are absorbed, and exert their biological effects, in a dose-dependent manner. Even from moderate doses (25 mL/d), which are lower than the traditional daily dietary intake in Mediterranean countries [34], around 98% of these phenolics are present in human plasma and urine in conjugated forms, mainly glucuronides and sulfate conjugates [35]. In addition, Perez-Maňa et al. [36] showed that Tyr is a precursor of HT endogenous formation in presence of ethanol, whose intake could enhance their bioavailability. After ingestion, EVOO polyphenols can be partially modified in the environment of the stomach. Aglycone secoiridoids are normally subjected to a time-dependent hydrolysis in the acidic gastric environment, which leads to a significant increase in the amount of free HT and Tyr after 30 min. This decomposition of secoiridoid aglycones increases with increased gastric residency, although under normal pH conditions (pH 2.0) and normal physiological time frames (up to 4 h) some remain intact and enter the small intestine un-hydrolyzed [37]. On the contrary, if the ingested secoiridoid is glycosilated, it is not subjected to gastric hydrolysis [38]. Therefore, phenolics such as the glucosides of oleuropein enter the small intestine unmodified, along with high amounts of free HT and Tyr. Following ingestion of EVOO, the levels of HT and Tyr increase rapidly achieving a peak concentration at approximately 1 h in human plasma and around 2 h in urine [35], which highlights that the small intestine is the major site of absorption for these compounds. 

The mechanism by which absorption occurs after EVOO polyphenols intake has been not completely highlighted. Manna et al. [39] studied for the first time the mechanisms of HT absorption using differentiated Caco-2 cell monolayers as a model system. They concluded that HT transport occurs via a bidirectional passive diffusion mechanism and estimated that the molecule was quantitatively absorbed at the intestinal level. Vissers et al. [38] suggested that the different polarities of the various phenolics might play a role in the absorption of these compounds. Moreover, it has been well established that, though absorption of EVOO phenolics in the gastrointestinal tract is good, bioavailability of these compounds in the ingested forms is poor due to an intensive metabolization of HT and Tyr at different levels [6,35,40]. Nevertheless, HT and Tyr might reach systemic concentrations useful to exert activities, other than free radical counteraction, by modulating key intracellular signals and improving enzymatic reactions catalyzed by various peroxidases and peroxiredoxins [41]. Once absorbed, HT and Tyr are widely distributed throughout the organism. The pharmacokinetic analysis in rats indicated an extensive and fast uptake of these compounds by different organs including skeletal muscle, kidneys, liver, lungs, and heart [42]. A recent study by Peyrol et al. [43] highlighted the importance of bilitranslocase in endothelial cell internalization of HT and its glucuronide metabolite. Moreover, it has been demonstrated that HT and its metabolites HT sulfate and HT acetate sulfate [40], as well as Tyr [44], are able to pass the blood brain barrier in rats and undergo brain uptake. 

### 2.3. Metabolism of Polyphenols 

**Figure 1:**
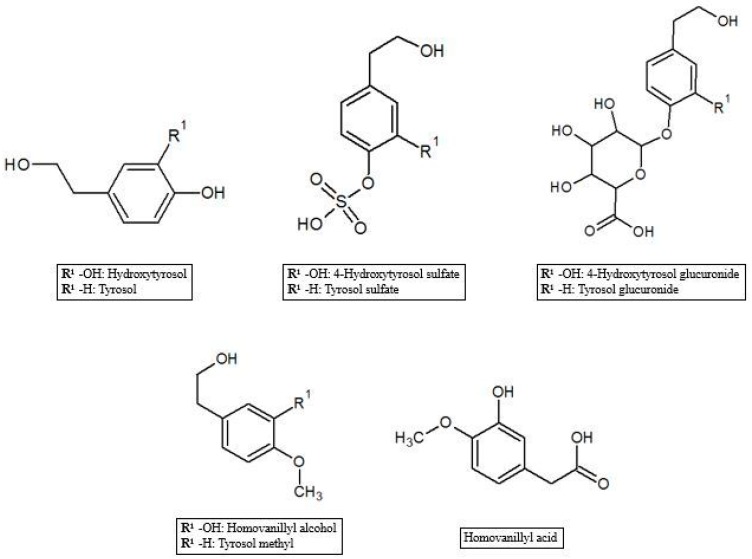
Hydroxytyrosol and tyrosol metabolites.

**Figure 2:**
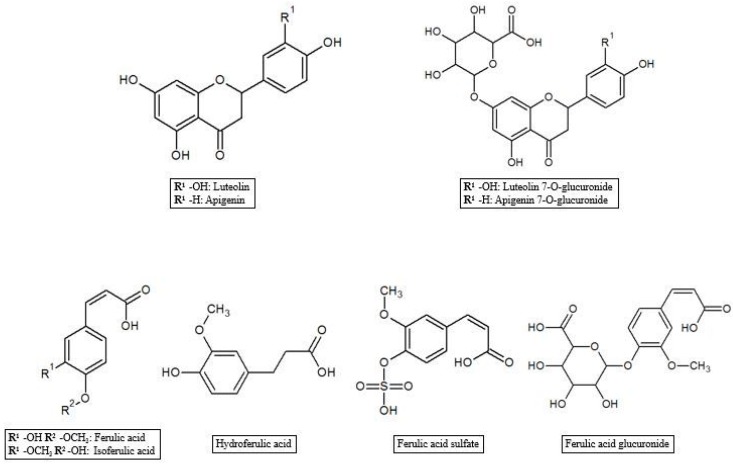
Luteolin, apigenin and ferulic acid metabolites.

As reported in the previous section, HT and Tyr metabolites are predominant in circulation after absorption in the gastrointestinal tract of the parent compounds (Figure 1). In the process of crossing enterocytes, EVOO phenolic compounds are subjected to an important first pass metabolism through a classic phase I/II biotransformation. This process is very relevant, to the extent that polyphenols in their un-metabolized form could be almost undetectable in biological matrices [45]. Indeed, more than 10 metabolites of HT and Tyr have been found in several animal and human studies. HT and Tyr are mainly subjected to three types of conjugation: methylation, glucuronidation and sulfation, through the respective action of catechol-O-methyl transferases (COMT), uridine-5′-diphosphate glucuronosyltransferases (UDPGT) and sulfotransferases (SULT) [46]. Thus, main metabolites include O-methylated forms [47], aldehydes and acids formed via oxidation of the aliphatic alcohol [42], sulfates [48], glucuronides [49], and acetylated and sulfated derivatives [50] as well as an N-acetylcysteine derivative [51]. The analysis of human plasma and urine has demonstrated that both HT and Tyr are dose-dependently absorbed and are metabolized primarily to O-glucuronidated conjugates (0.67–0.95 µmol in 24 h collected urines after 50 mL EVOO intake) [47,49,52,53]. In fact, sulfated and glucuronidated HT and Tyr were the predominant metabolites found in human plasma and urine, and they have also been shown to concentrate in the intestinal epithelium, since glucuronidation and sulfation are the major pathways of phase II xenobiotic metabolism in the human intestine [35]. Recent data on HT bioavailability demonstrated that glucuronide (73–74 nmol/L) [51] and sulfate (34 nmol/L) [51] metabolites appear to be the most abundant among the HT phase II metabolites in rat urine [36,51,54]. Another important metabolic pathway of HT is known to rise to the formation of 3-hydroxy-4-methoxyphenylethanol (homovanillyl alcohol) in a reaction regulated by the catecholmethyltransferase (COMT) [55]. Moreover, HT, homovanillyl alcohol, and homovanillic acid are endogenously formed by dopamine oxidative metabolism and might be found in biological matrices though at low concentrations [6]. Furthermore, Rubio et al. [56] showed that the main phenolic metabolites detected in human plasma samples 0–6 h after ingestion of EVOO were HT sulfate (264–764 µmol/min AUC), HT acetate sulfate (85.5–383 µmol/min AUC), homovanillic acid (38.2–120 µmol/min AUC) and homovanillic acid sulfate (12.7–79.5 µmol/min AUC), while Kountouri et al. [57] found in human urines relative high amounts of 3,4-dihydroxyphenylacetic acid (14.0 µg/mL), homovanillyl alcohol (0.12 µg/mL) and homovanillic acid (2.2 µg/mL) in addition to the aforementioned metabolites. Soler et al. [28] showed instead that HT is metabolized in intestinal cells as sulfate, methyl (homovanillyl alcohol) and methyl sulfate (homovanillyl alcohol sulfate) while Tyr, because of the absence of an –OH group with respect to HT chemical structure, originated only sulfate and methyl metabolites. Glucuronide metabolites were instead observed for minor components such as luteolin (luteolin glucuronide, 4.48%; and luteolin methyl glucuronide, 4.27% of total metabolites after olive oil intake), pinoresinol (pinoresinol glucuronide, 6.09% of total metabolites after olive oil intake) [28,58], and ferulic acid (ferulic acid glucuronide, 14.9%; sulfate, 10.9%, and sulfoglucuronide, 69.7 % of total ferulates after ferulic acid intake) (Figure 2) [59]. 

## 3. Activity of Polyphenols Metabolites

### 3.1. Hydroxytyrosol and Tyrosol Glucuronides and Sulfates 

In the last 15 years the attention on sulfate and glucuronide metabolites when assessing in vitro and in vivo experiments has increased among researchers (Table 1), though there are still question marks about the actual effectiveness of such compounds. Indeed, several research groups pointed out that sulfate and glucuronide moieties can be deconjugated once they get intracellular environment, thus actually acting as free parent compounds and not as metabolites [50]. For example, Peyrol et al. [43] showed that HT glucuronide exerted the same antioxidant activity of HT in endothelial cells: however, this was due to the intracellular β-glucuronidase action which favored deconjugation, leading to the formation of free HT. Moreover, Atzeri et al. [60] observed that HT and Tyr sulfate, entered intestinal Caco-2 cells from 30 min of incubation and underwent an extensive metabolization, giving rise to a pool of metabolites, mainly sulfate and methyl-sulfate HT and Tyr.

Thus, it is conceivable that, though deconjugated before entering the cells, HT and Tyr metabolites can be reformed inside the cell environment so acting as conjugated forms together with HT and Tyr free forms. In literature different opinions about loss/increase of activity of HT and Tyr, due to the presence of the sulfate/glucuronide functional groups, are still debated. For instance, Khymenets et al. [51,61] highlighted a loss of antioxidant activity of HT 3’-O- and 4’-O-glucuronides and Tyr 4’-O-glucuronide. Such results were in line with a previous theoretical study in which phase II metabolites glucuronides (and sulfates) were predicted to lose the antiradical activity characteristic of their parent compounds [62]. In contrast, one of our studies [63] reported that HT glucuronide metabolites, specifically the mix of 3’-O-ß-D and 4’-O-ß-D-glucuronidated isoforms, could protect renal cells (LLC-PK1 cells as a culture model) against H_2_O_2_-induced lipid peroxidation-related membrane oxidative damage. In any case, glucuronide metabolites were less active than HT free form. In a different study, HT glucuronides were shown to protect red blood cells (RBC) from in vitro H_2_O_2_-induced oxidative injury at low concentrations [64]. At higher concentrations, however, the protective effect of HT glucuronides did not increase. The authors stated that a saturation of the glucuronide-specific RBC transporters could have occurred, limiting the availability of glucuronides inside RBC, or/and the glucuronides may have undergone a restricted hydrolysis to liberate active HT, which could then be absorbed by the cells. Interestingly, a report of Atzeri et al. [60] evaluating the antioxidant effect of HT and Tyr sulfate metabolites in intestinal cells (Caco-2) found that they displayed an efficiency comparable to that of the parent compounds. Moreover, HT and Tyr sulfates showed anti-inflammatory and antioxidant activities in HUVEC preventing the rise of reactive oxygen species, the depletion of glutathione, and the down-regulation of glutathione peroxidase 1, glutamate-cysteine ligase catalytic subunit, and heme oxygenase-1 genes [65]. Tyr sulfate and glucuronide were also able to prevent the phosphorylation of NF-κB signaling proteins, as well as the over-expression of adhesion molecules at gene, protein, and secretory levels, and the adhesion of human monocytes to the endothelial cells [65]. Still regarding the protective effects at cardiovascular level, Giordano et al. [66] established that two different HT glucuronide metabolites, 3-O-HT glucuronide and 4-O-HT glucuronide, were able to inhibit tunicamycin-induced endoplasmic reticulum (ER) stress, which is supposed to be relevant to atherosclerosis onset and development. 

Compared with the effects of HT, 3-O-HT glucuronide and 4-O-HT glucuronide at physiological concentrations of 10 μM and 25 μM, induced in vitro a milder change in mRNA expression levels of both CCAAT-enhancer-binding protein homologous protein (CHOP) and glucose regulated protein GRP78 immunoglobulin heavy chain binding protein (BiP). Moreover, a mix of HT phase-II metabolites (HT sulfate, HT glucuronide, homovanillic alcohol sulfate, homovanillic alcohol glucuronide) was used to evaluate the effectiveness in attenuating the initial steps of atherosclerosis using a translational approach with in vitro and in vivo experiments [67]. Specifically, that study highlighted the ability of EVOO phenols to reduce VCAM-1, ICAM-1, E-selectin and MCP-1 molecules secretion in mice aortas by inhibiting mRNA expression. The same mix of HT metabolites reduced the adhesion of the lymphocytes to aortic cells (HAECs) at all concentrations tested, with significantly greater reductions with HT metabolites than free HT. Protein and mRNA reduction by HT metabolites appeared to be regulated by the MAPK pathway but not by the NF-κB pathway. In fact, HT metabolites significantly reduced the phosphorylation of p38δ, JNK1-3, CREB, AKT3, p53 and P70 S6 Kinase. 

As regards the modulation of brain functions, HT, HT sulfate and HT acetate sulfate have recently been the focus of an investigation lead by López de las Hazas et al. [68], where their protective effects against oxidative stress at physiological concentrations (10 μM) in neuroblastoma (SH-SY5Y) and dopaminergic (LUHMES) neuronal cells were ascertained. This aspect is biologically relevant because of the proven accumulation of these compounds in the brain, where they may reach concentrations useful to achieve positive effects [40,68]. 

### 3.2. Homovanillic Acid and Homovanillyl Alcohol

Among the main HT metabolites which are bioavailable at biological relevant concentrations, homovanillic acid and homovanillyl alcohol have been largely studied in different experimental systems. It is well established that homovanillic acid and homovanillyl alcohol possess a good antioxidant and antiradical capacity [48], though as ortho-methoxy phenols are less effective than simple ortho-phenols such as HT [69]. Nevertheless, depending on the experimental model used, the antioxidant activity of homovanillyl alcohol resulted lower [70,71,72] or higher [73] than that of HT. In any case, homovanillyl alcohol is chemically more stable in biological fluids than HT [74], therefore its activity might be exerted for a longer time. In relation to a protective role at gastrointestinal level, we showed in Caco-2 cells monolayers that HT, Tyr and homovanillyl alcohol were able to protect cell membranes from oxidative damage induced by tert-butil hydroperoxide (TBH) treatments [75]. The mechanism of action was related to a direct scavenging of peroxyl radicals and a hydrogen atom-donating activity [75]. Moreover, homovanillyl alcohol inhibited, to a lesser extent with respect to HT, NF-kB driven transcription and nuclear translocation in human gastric adenocarcinoma (AGS) cells, used as in vitro model of gastric inflammation [76]. 

As regards vascular regulation, an interesting clinical study of De la Torre et al. [55] pointed out for the first time the association between high urinary homovanillyl alcohol concentrations and a lower risk of CVD and total mortality in elderly individuals at a high risk of CVD. Among the mechanisms involved in the beneficial effects on cardiovascular system, in vitro models demonstrated that homovanillyl alcohol was able to inhibit cell surface expression in endothelial HUVEC cells of E-selectin, ICAM-1 and VCAM-1 adhesion molecules, which are crucial for endothelial activation and therefore for the onset of atherosclerosis, whereas the effect on mRNA expression was evident only for E-selectin [77]. This outcome was confirmed by another study on HUVEC cells of Turner et al. [73], where ICAM-1, VCAM-1 and MCP-1 secretion was inhibited by HT and homovanillyl alcohol, and by Manna et al. [78], where homovanillyl alcohol reduced cell adhesion and ICAM-1 expression in human monocytic cells U937. In human erythrocytes, it was instead reported that both HT and homovanillyl alcohol significantly decrease ROS-induced oxidative stress at 10–50 µM, and HT was more active than homovanillyl alcohol, likely due to its better scavenging and iron chelating activities [79]. Moreover, HT together with homovanillyl alcohol, homovanillyl alcohol glucuronide, homovanillyl alcohol acetate and other metabolites protected RBC in a significant way from oxidative-AAPH (dihydrochloride) induced hemolysis at the concentration of 80 μM. At lower concentrations (20−60 μM), the same compounds, except homovanillyl alcohol glucuronide, protected RBCs from oxidative hemolysis in a dose-dependent manner. Indeed, the homovanyllyl alcohol glucuronide showed the weakest effect among the tested compounds [64]. A recent study by Serra et al. [80] assessed in peripheral mononucleated blood cells (PBMCs), showed that homovanillyl alcohol, although less than HT, was able to inhibit oxysterols-induced production of pro-inflammatory cytokines, interleukin-1β, regulated on activation, normal T-cell expressed and secreted and macrophage migration inhibitory factor. Moreover, it was effective in contrasting the increase of ROS and phosphorylation of the p38 and JNK MAPK [80]. Other beneficial activities were observed at kidney level, where in LLC-PK1 cells homovanillyl alcohol, as well as HT, proved to protect against H_2_O_2_-induced injury, at least in part through the modulation of proteins involved in the cellular signaling pathways, as Akt and MAPK ERK, and JNK [70].

### 3.3. Other Polyphenol Metabolites

More than 30 phenolic compounds might be found in EVOO beyond the renowned HT and Tyr, whose metabolites have been well talked over in the previous sections. There are just few papers debating healthy properties of the metabolites coming from minor component of EVOO phenolic fraction. Among them, one of the most studied is ferulic acid (FA), whose metabolism in vivo is known to produce isomers (such as isoferulic acid) or conjugated molecules such as FA glucuronide and sulfate [59,81]. It has been reported that FA sulfate, better than FA, is able to elicit a concentration-dependent vasorelaxation of isolated mouse saphenous and femoral arteries and aorta. In anesthetized mice, intravenous injection of FA-sulfate decreased mean arterial pressure, whereas FA had no effect, FA-sulfate is then probably one of the major metabolites accounting for the blood pressure-lowering effects associated with FA intake [82]. Isoferulic acid proved instead to be a multifunctional compound, showing antiviral [83], anti-inflammatory [84], antioxidative [84] and antidiabetic properties [85]. Quite recently, isoferulic acid also showed antiglycation properties against fructose and glucose-mediated glycation and oxidation of bovine serum albumin [86]. Another isomer, hydroferulic acid, has proved to be an inhibitor of in vitro platelet activation more effective than its phenolic precursor FA [87]. 

Some other recent studies report the biological activity of metabolites originated by the minor polar components luteolin and apigenin. A study by Ha et al. [88] interestingly found that luteolin was more effective as anti-inflammatory agent in LPS stimulated Raw 264.7 cells as glucuronide and sulfate metabolite rather than as methyl metabolite. In particular, expression of iNOS and production of nitric oxide and pro-inflammatory cytokines such as TNF-α, IL-1β, and IL-6 were better suppressed by those compounds which were metabolized in hepatic HepG2 only as glucuronide and sulfate, while the same metabolites which subsequently underwent COMT metabolization did not exert the same activity. Inflammatory conditions led by LPS treatments in isolated human neutrophils showed instead the activation of β-glucuronidase, which deconjugated luteolin to produce its free form [89]. This can indicate that luteolin metabolites in the first study could have been effective both as luteolin free form and as a mix of different metabolites, depending on their concentrations once released and on deconjugation levels. In fact, Kure et al. [90] found that different luteolin glucuronides (3-O-glucuronide and 7-O-glucuronide) were effective in reducing expression of inflammatory genes in LPS-treated RAW264.7 cells, but the effects of luteolin free form was predominant. The anti-inflammatory effects of a luteolin metabolites pool were confirmed also by Ma et al. [33], who found luteolin 7-O-glucuronide as the most effective in limiting LPS-induced inflammation in RAW264.7 cells. The same activities were also observed in the same cell line treated with another EVOO metabolite which is similar in structure to luteolin 7-O-glucuronide, apigenin-7-O-glucuronide [91,92], which showed to be effective in counteracting prostaglandin E2 (PGE2) production, LPS-induced mRNA expression of iNOS, cyclooxygenase-2 (COX-2), and TNF-α. Moreover, treatments with apigenin-7-O-glucuronide decreased the translocation of c-Jun into the nucleus, and decreased activator protein-1 (AP-1)-mediated luciferase activity through the inhibition of both p38 and extracellular signal-regulated kinase (ERK) phosphorylation. Another study reported apigenin-7-O-glucuronide to be also effective in inhibiting modified low-density lipoprotein uptake and the formation of foam cells, so acting as anti-atherosclerotic agent [32]. In addition, it resulted to be able to inhibit the scavenger receptor CD36 mRNA and protein expression, and to enhance the expression of scavenger receptor class B type 1, by inhibiting ERK1/2 phosphorylation [32].

## 4. Conclusions and Future Research

Most bioavailability studies conducted so far agree that plasma and tissues concentration of EVOO polyphenols metabolites is often higher than the concentration reached by the ingested parent compounds. Thus, these metabolites are likely to significantly contribute to the beneficial health effect correlated to the regular consumption of olive products. However, although in the last decade several studies have been focused on the evaluation of the potential health benefits of EVOO phenols metabolites, the data available are still limited. In vitro studies demonstrated that metabolites are able to exert beneficial effects others than antioxidant and scavenging properties, by modulating intracellular signaling. At cardiovascular and gastrointestinal level in particular, metabolites showed the ability to ameliorate physiological condition and to prevent exacerbation of inflammation and oxidative stress, with an efficiency comparable or even greater than that of the parent compounds. It was also observed in many cases that metabolites undergo deconjugation before entering the cells, releasing free forms which are partially converted into other metabolites. To understand the impact of metabolites on human health, next studies regarding principal EVOO phenols (namely HT and Tyr) and minor compounds must be planned to focus on what is really close to real biological conditions, with particular attention on concentrations and composition of EVOO metabolites at different body levels. Nevertheless, acquired information, mainly in in vitro experimental models, suggests a significant biological role of these metabolites. They represent the largest part of a continually changing pool of compounds, which forms following polyphenols ingestion, that is probably responsible, as a whole, for the observed beneficial effect in the prevention and amelioration of the major degenerative diseases.

## Figures and Tables

**Table 1:** Olive oil polyphenols metabolites and their activities in different in vitro and in vivo experimental models.

Compounds	Concentration Tested	In Vitro/In Vivo Model	Outcome	Reference
Mix of metabolites	10–80 µM	In vitro red blood cells (RBC)	Protection of red blood cells from H_2_O_2_-induced oxidative injury	[64]
	10 mg/kg	ApoE−/− mice	Reduction of VCAM-1, ICAM-1, E-selectin and MCP-1 molecules secretion by inhibiting mRNA expression	[67]
	1–5 μM	Human aortic endothelial cells (HAEC)	Reduction of p38δ, JNK1-3, CREB, AKT3, p53 and P70 S6 kinase phosphorylation, and of lymphocytes adhesion	[67]
HT glucuronide	100 μM	Rat aortic rings	Reduced endothelial dysfunction by blocking superoxide production	[43]
	2.3 µM	DPPH test	Good antioxidant and antiradical capacity	[48]
	0.01–10 μM	In vitro Cu-induced oxidation of LDL	Loss of antioxidant activity with respect to HT	[61]
	5–10 μM	Renal LLC-PK1 cells culture model	Protection of renal cells against H_2_O_2_-induced lipid peroxidation	[63]
	10–25 µM	Human hepatocarcinoma HepG2 cells	Inhibition of tunicamycin-induced endoplasmic reticulum (ER) stress	[66]
HT sulfate	91 µM	DPPH test	Poor antioxidant and antiradical capacity	[48]
	2.5–10 µM	Caco-2 intestinal cells monolayers	Counteraction of the oxidizing action of oxidized cholesterol on intestinal cell membranes	[60]
	10 µM	Neuroblastoma (SH-SY5Y) and dopaminergic (LUHMES) neuronal cells	Protective effects against oxidative stress	[68]
Tyr glucuronide	0.01–10 μM	In vitro Cu-induced oxidation of LDL	Loss of antioxidant activity with respect to Tyr	[61]
	100 µM	Endothelial HUVEC cells monolayers	Prevention of the phosphorylation of NF-κB signaling proteins and of the over-expression of adhesion molecules	[65]
	0.1–0.5 mg/kg	Carrageenan-induced hind paw oedema in mice	Amelioration of plantar and ear edemas	[65]
Tyr sulfate	2.5–10 µM	Intestinal Caco-2 cells monolayers	Counteraction of the oxidizing action of oxidized cholesterol on intestinal cell membranes	[60]
	0.1–0.5 mg/kg	Carrageenan-induced hind paw oedema in mice	Amelioration of plantar and ear edemas	[65]
	100 µM	Endothelial HUVEC cells monolayers	Prevention of the phosphorylation of NF-κB signaling proteins and of the over-expression of adhesion molecules	[65]
Homovanillic acid	14.8 µM	DPPH test	Good antioxidant and antiradical capacity	[48]
Homovanillyl alcohol	11.4 µM	DPPH test	Good antioxidant and antiradical capacity	[48]
	5.4–146.5 mM	Human clinical study	Reduction of CVD and total mortality risk	[55]
	0.3–1 µM	Renal LLC-PK1 cell culture model	Protection against H_2_O_2_-induced renal epithelial injury through interaction both MAP kinase and PI3 kinase pathways	[70]
	2–20 µM	Endothelial HUVEC cells monolayers	Inhibition of ICAM-1, VCAM-1 and MCP-1 secretion	[73]
	5–25 µM	Intestinal Caco-2 cells monolayers	Protection of cell membranes from oxidative damage induced by TBH	[75]
	0.5–10 mM	Human gastric adenocarcinoma (AGS) cells	Inhibition of NF-kB driven transcription and nuclear translocation	[76]
	0.5–25 µM	Endothelial HUVEC cells monolayers	Inhibition of cell surface expression of E-selectin, ICAM-1 and VCAM-1 adhesion molecules	[77]
	1–7.5 µM	Human monocytic cells U937	Reduced cell adhesion and ICAM-1 expression	[78]
	10–50 µM	Erythrocytes by blood samples obtained from trisomic patients	Decreased oxidative stress-induced ROS generation	[79]
	0.25–1 µM	Peripheral mononucleated blood cells (PBMCs)	Inhibition of oxysterols-induced production of proinflammatory cytokines, interleukin-1β, normal T-cell macrophage migration inhibitory factor. Decreased levels of reactive oxygen species (ROS) and phosphorylation of the p38 and JNK MAP kinases	[80]
FA glucuronide	11.42–114.2 µg/kg	Male Swiss mice	Elicitation of vasorelaxation of saphenous and femoral arteries and aortae. Decreased mean arterial pressure	[82]
IsoFA	0.5–1 mg/day	Mice infected by intranasal inoculation of influenza virus	Inhibition of the progression of lethal influenza virus pneumonia	[83]
	1–13 μg/mL	In vitro lipid peroxidation, DPPH and ABTS tests	Good antioxidant activity	[84]
	5.0 mg/kg	Streptozotocin-induced diabetic rats	Inhibition of hepatic gluconeogenesis and increase of the glucose utilization in peripheral tissue to lower plasma glucose	[85]
	1.25–5 mM	In vitro Glycation of Bovine Serum Albumin (BSA)	Antiglycation properties against fructose and glucose-mediated glycation and oxidation of bovine serum albumin	[86]
HydroFA	0.01–100 μg/mL	In vitro platelet culture	Inhibitor of platelet activation by decreasing P-selectin expression	[87]
Luteolin sulfate	40 μM	Macrophages Raw 264.7 cells	Anti-inflammatory activities as inhibition of LPS-stimulated iNOS expression and production of nitric oxide, TNF-α, IL-1β, and IL-6	[88]
Luteolin glucuronide	25–200 μg/mL	Macrophages Raw 264.7 cells	Anti-inflammatory activities as inhibition of LPS-stimulated iNOS expression and production of nitric oxide, TNF-α, IL-1β, and IL-6	[33]
	40 μM	Macrophages Raw 264.7 cells	Anti-inflammatory activities as inhibition of LPS-stimulated iNOS expression and production of nitric oxide, TNF-α, IL-1β, and IL-6	[88]
	50 mM	Macrophages Raw 264.7 cells	Reduction of expression of LPS-stimulated inflammatory genes	[90]
Apigenin glucuronide	25–100 μg/mL	Macrophages Raw 264.7 cells	Inhibition of Ox-LDL uptake and the scavenger receptor CD36 mRNA and protein expression. Enhancement of the expression of scavenger receptor class B type 1, following inhibition of ERK1/2 phosphorylation	[32]
	12.5–100 µM	Macrophages Raw 264.7 cells	Counteraction of prostaglandin E2 (PGE2) production, LPS-induced mRNA expression of iNOS, COX-2 and TNF-α Decrease of the translocation of c-Jun into the nucleus inhibition p38 and ERK phosphorylation	[91]
